# Association Between Dementia and Optical Coherence Tomography Scan Quality

**DOI:** 10.14336/AD.2024.1744

**Published:** 2026-02-13

**Authors:** Reuben Jyong Kiat Foo, Damon Wong, Nur Fidyana Binte Abdul Gani, Bingyao Tan, Munirah Binte Ismail, Gerhard Garhöfer, Laetitia Hinterhuber, Narayanaswamy Venketasubramanian, Christopher Li-Hsian Chen, Leopold Schmetterer, Jacqueline Chua

**Affiliations:** ^1^Singapore Eye Research Institute, Singapore National Eye Centre, Singapore, Singapore.; ^2^SERI-NTU Advanced Ocular Engineering (STANCE), Singapore, Singapore.; ^3^School of Chemistry, Chemical Engineering and Biotechnology, Nanyang Technological University, Singapore, Singapore.; ^4^Department of Clinical Pharmacology, Medical University Vienna, Wien, Austria.; ^5^Institute of Artificial Intelligence, Center for Medical Data Science, Medical University Vienna, Wien, Austria.; ^6^Memory Aging and Cognition Centre, Departments of Pharmacology and Psychological Medicine, Yong Loo Lin School of Medicine, National University of Singapore, Singapore, Singapore.; ^7^Raffles Neuroscience Centre, Raffles Hospital, Singapore, Singapore.; ^8^Center for Medical Physics and Biomedical Engineering, Medical University Vienna, Wien, Austria.; ^9^Ophthalmology and Visual Sciences Academic Clinical Program, Duke-NUS Medical School, National University of Singapore, Singapore, Singapore.; ^10^Aier Hospital Group, Changsha, China.; ^11^Fondation Ophtalmologique Adolphe De Rothschild, Paris, France.

**Keywords:** Alzheimer’s disease;, dementia, mild cognitive impairment, optical coherence tomography, scan quality

## Abstract

It is generally assumed that dementia affects the quality of optical coherence tomography (OCT) scans. However, the magnitude of this effect and its independence from other factors require further clarification. In this cross-sectional study, our aim was to evaluate the association between cognitive impairment and OCT scan quality, adjusting for key confounders, in a multiethnic cohort. 541 participants aged 50 years or older were recruited from memory clinics and the community at the National University Hospital and St. Luke’s Hospital, Singapore. They were then stratified into three groups: no cognitive impairment (NCI, n=112), cognitive impairment without dementia (CIND, n=235), and dementia (n=194); OCT scan quality was subsequently assessed based on the presence and severity of artifacts. We found that dementia patients were nearly three times more likely to produce poor-quality OCT scans compared to NCI participants (adjusted odds ratio [OR]=2.90; 95% CI, 1.24-6.80). Lower cognitive scores, including Mini-Mental State Examination (MMSE) (OR=0.92; 95% CI, 0.88-0.96), Montreal Cognitive Assessment (MoCA) (OR=0.90; 95% CI, 0.86-0.94), and higher Clinical Dementia Rating (CDR) scores (OR=2.11; 95% CI, 1.43-3.10), were also independently associated with poor scan quality. In conclusion, cognitive impairment, particularly dementia, substantially increases the likelihood of poor-quality OCT scans, even after accounting for key demographic and clinical factors. Hence, strategies tailored to improve imaging in this population are essential for enhancing diagnostic accuracy and patient care.

## INTRODUCTION

Optical coherence tomography (OCT) is a critical imaging tool in ophthalmology, offering high-resolution images of the retina and optic nerve [1]. While OCT is indispensable for diagnosing and monitoring various ocular diseases, scan quality is critical for accurate interpretation. Poor-quality scans, often caused by artifacts, can lead to misdiagnoses, including masking disease progression or generating false-positive findings [2]. Although factors such as ocular pathologies [2] and older age [2, 3] are known to influence scan quality, the role of cognitive impairment, particularly dementia, remains insufficiently studied.

Dementia, a prevalent neurodegenerative disease, is associated with difficulties in tasks requiring patient cooperation, including imaging procedures. This has led to the assumption that poor-quality OCT scans are more common among dementia patients. With the rising prevalence of dementia [4] and its associated increased risk of eye diseases [5], understanding the impact of cognitive dysfunction on OCT scan quality is critical. While reduced cognitive function has been linked to challenges with imaging modalities like OCT angiography [6], the specific relationship between dementia and the prevalence of artifacts in OCT scans, independent of key confounders, remains poorly defined.

This study seeks to address this gap by evaluating the association between cognitive impairment and OCT scan quality of the optic disc in a multiethnic cohort. By adjusting for key confounders such as age, sex, race, education, and hypertension, this study provides robust insights into how dementia influences scan quality. These findings have significant clinical implications, helping clinicians identify patients at risk for poor-quality scans and adapt OCT procedures to improve diagnostic accuracy and disease monitoring in this vulnerable population.

## MATERIALS AND METHODS

### Study Participants

This cross-sectional study was conducted with approval from the National Healthcare Group Domain-Specific Review Board (NHG DSRB reference number 2018/01098 and 2010/00017), and in accordance with the Declaration of Helsinki. Written informed consent was also obtained from all participants or their caregivers prior to recruitment. As described in previous studies [5, 7, 8], individuals aged 50 and older were recruited from the National University Hospital of Singapore and St. Luke’s Hospital from September 2009 to September 2020. They also met the following criteria:

a) Had sufficient language skills for neuro-psychological assessment (in English, Mandarin, or Malay).
b) Cognitive impairment was not due to potentially reversible causes, for instance thyroid disorders, vitamin B12 deficiency, and other treatable conditions.
c) Did not have a history of substance abuse disorder, major psychiatric illness, cognitive impairment due to traumatic brain injury, multiple sclerosis, epilepsy, tumors, or significant visual or auditory abnormalities.
d) Did not have significant aphasia or dysarthria that would preclude cognitive assessment

For diagnosis of cognitive impairment, a locally-validated neuropsychological test battery (Vascular Dementia Battery [VDB] [9]) was administered by trained research psychologists. The VBD assesses across six cognitive domains, namely attention, language, verbal memory, visual memory, visuoconstruction, and visuomotor speed. Based on this, the following diagnostic groups were classified:

- Controls with no cognitive impairment (NCI) were recruited from both memory clinics and the community and had no objective impairment in any of the domains of the VDB.
- Participants with cognitive impairment and no dementia (CIND) were defined as those who had objective impairment in one or more domains of the VDB without loss of daily functions, but failed to meet the Diagnostic and Statistical Manual of Mental Disorders (DSM-IV) criteria for dementia [10].
- Dementia participants were diagnosed based on criteria from the DSM-IV [11].
- Within the dementia group, Alzheimer’s disease (AD) was diagnosed using the National Institute of Neurological and Communicative Disorders and Stroke and the Alzheimer's Disease and Related Disorders Association (NINCDS-ADRDA) [12], and vascular dementia was diagnosed using the National Institute of Neurological Disorders and Stroke and Association Internationale pour la Recherché et l' Enseignement en Neurosciences (NINDS-AIREN) criteria [13].

Three brief cognitive tests were administered by trained research psychologists, namely the Mini-Mental State Examination (MMSE), the Montreal Cognitive Assessment (MoCA), and the Clinical Dementia Rating (CDR). MMSE and MoCA are dementia screening instruments with a maximum score of 30, and higher scores indicate better cognition [14]. Conversely, the CDR global score ranges from 0 to 3, with higher scores indicating more severe symptoms of dementia [15].

### Data Collection

Patient demographics (age, sex, race, years of education) and medical history (hyperlipidemia, hypertension, diabetes) were collected via interviewer-administered questionnaires and verified against medical records. Blood pressure (BP) was measured twice after subjects were seated for 5 minutes, with a third reading taken if variations exceeded 10 mmHg (systolic) or 5 mmHg (diastolic).

Participants were excluded if they had conditions affecting the central nervous system (CNS), including hypoxic, anoxic, hypotensive, hypertensive, uremic, or hepatic encephalopathy; traumatic, nutritional, or toxic CNS disorders; substance abuse impacting the CNS; intracerebral hemorrhage; cranial arteritis; CNS inflammatory vasculitis; moyamoya disease; CNS infection; intracranial mass lesions; obstructive or normal pressure hydrocephalus; uncontrolled epilepsy; or medical conditions requiring corticosteroid or immunosuppressant therapy. Additionally, those in a moribund state or with significant aphasia or dysarthria impeding cognitive assessment were excluded.

All participants underwent standardized eye examinations, including auto-refraction-keratometry (Canon RK-5 Autorefractor Keratometer; Canon Inc., Tokyo, Japan) and measurement of intraocular pressure (IOP). Ocular assessments were performed within a month from the cognitive assessments, using fundus photographs taken with a digital retinal camera (Canon CR-1 Mark II; Canon Inc., Tokyo, Japan) after pupil dilation with 1% tropicamide. A trained grader masked from participants’ characteristics evaluated the fundus photographs for retinal diseases [5, 16], such as age-related macular degeneration (AMD), diabetic retinopathy (DR), glaucoma, and other retinal disorders (e.g., vascular occlusions, macular holes, and chorioretinopathy). Photographs obscured by more than a quarter were considered ungradable.

Structural OCT imaging using the Zeiss Cirrus HD-5000 Spectral-Domain OCT (Carl Zeiss Meditec, Dublin, CA). Each eye was imaged using a 200 A-scan × 200 B-scan (6 × 6 mm²) protocol centered on the optic disc. FastTrac™ motion correction (20 Hz line-scanning ophthalmoscope) was employed to minimize artifacts from motion and blinks. Technicians received rigorous training and followed standardized procedures to ensure consistent, high-quality scans. Spherical equivalent was calculated by summing the spherical value and half the negative cylinder value. Scan signal strength and retinal nerve fiber layer (RNFL) thickness measurements were obtained using Cirrus Review Software (version 11.0.0.29946).

### OCT scan quality and artifacts

**Figure 1.**
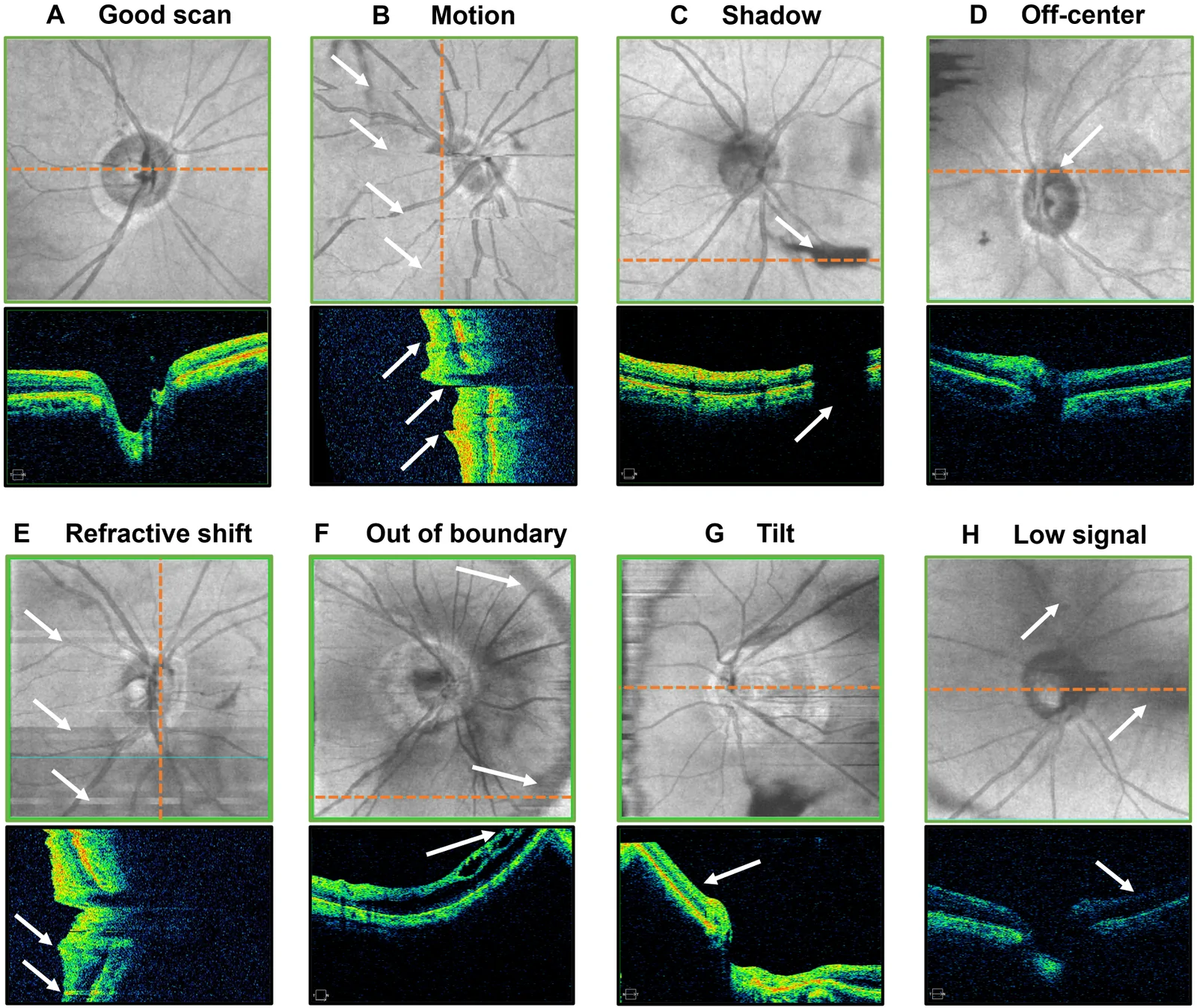
**Examples of artifact types encountered during optical coherence tomography (OCT) imaging**. The figure includes both good-quality (A) and poor-quality (B-H) enface scans, with corresponding cross-sectional B-scans for poor-quality examples. White arrows indicate the location of the artifact, and orange dotted lines mark the location of the B-scan. Panels show: (A) Good-quality scan; (B) Motion artifact; (C) Shadow artifact; (D) Off-center artifact; (E) Refractive shift; (F) Out-of-boundary artifact; (G) Tilt artifact; (H) Low-signal artifact.

Structural OCT scans were reviewed by a single trained grader blinded to participant characteristics. Scans were assessed for artifact types (Fig. 1), including:

- Good scan (Fig. 1A): These scans were well-centered, with the optic disc in focus and retinal layers clear and evenly visualized. Such scans were characterized by the absence of artifacts or artifacts that did not affect the region of measurement interest. Signal strength was ≥ 6, and segmentation was consistent across the scan.
- Motion (Fig. 1B): Defined as horizontal discontinuity or waviness of retinal vessels and structures due to subject movement. Motion artifacts were severe if they occurred within the circumpapillary ring and caused severe discontinuities (e.g., vessels becoming fully disconnected). Breaks outside the circumpapillary ring or very slight breaks within the ring that maintained continuity were considered acceptable.
- Shadow (Fig. 1C): Caused by ocular opacities leading to low signal and light blockage. Shadow artifacts were severe if they had high opacity (i.e., loss of signal across all retinal layers) and were within the circumpapillary ring or covered a large area (>10%). Minor shadows outside the measurement region were acceptable.
- Off-center (Fig. 1D): Also referred to as decentration, this occurred when the scan was not centered on the optic disc. Severe off-center artifacts had the optic disc displaced from the image center by more than 10% of the imaging field-of-view. Slight (<1%) to moderate (1-10%) off-center artifacts were acceptable.
- Refractive shifts (Fig. 1E): Represented as alteration in intensity between adjacent scans, often due to blinking or changes in the corneal surface refractive index [17]. Severe refractive shifts resulted in significant B-scan signal reduction or complete signal loss across most or all retinal layers and occurred within the circumpapillary ring. Minor refractive shifts outside critical regions were acceptable.
- Out of Boundary (Fig. 1F): Also referred to as out-of-window or out-of-register artifacts, these occurred when B-scans were cut off at the top or bottom, resulting in incomplete imaging of retinal layers [18, 19]. Severe out-of-boundary artifacts affected the circumpapillary ring or constituted a large area of the enface scan (>10%). Artifacts outside the measurement region were acceptable.
- Tilt (Fig. 1G): Poor alignment leading to severe retinal tilt in the B-scan, causing part of the image to be low-signal or out of focus [20, 21]. Severe tilt artifacts had only half or less of the scan in focus, with poor contrast in the out-of-focus region. Moderate tilt that maintained good contrast across the entire image was acceptable.
- Low signal (Fig. 1H): Occurred when the scan’s signal strength was insufficient (signal strength < 6), leading to unclear and noisy visualization of retinal layers. Severe low-signal artifacts caused inconsistent intensity and obscured retinal structures.

### Statistical analysis

In this study, participants with poor-quality scans in both eyes (the 'excluded' group) were compared to those with good scans in at least one eye (the 'included' group). Participants were considered to have ocular diseases if at least one eye had an ocular disease present. This comparison aimed to identify factors associated with poor-quality scans, which could help pinpoint individuals at higher risk of imaging challenges.

Group differences (NCI, CIND, dementia) were analyzed using chi-squared tests for categorical variables and Kruskal-Wallis or analysis of variance (ANOVA) for continuous variables. The Shapiro-Wilk test was used to determine normality. Univariate and multivariable-adjusted logistic regression models were used to determine odds ratios (ORs) and 95% confidence intervals (CIs) for associations between cognitive diagnoses or tests (predictor) against OCT scan quality (outcome). Confounders identified from univariate regression, such as age, sex, race, education, and hypertension, were subsequently adjusted for in the multivariate regression models. To assess the prevalence of artifact types, we considered a participant to have an artifact if it was present in either of their eye scans. Statistical analyses were performed using SPSS Statistics version 26 (IBM, USA), and p-values below 0.05 were considered significant.

## RESULTS

A total of 541 study participants received OCT imaging. Of these, 112 (21%) were diagnosed with NCI, 235 (43%) with CIND, and 194 (36%) with dementia. Table 1 shows the clinical characteristics of the participants stratified by neurological diagnosis. CIND and dementia participants were older (median age (IQR) = 75 (9) years and 75 (10) years respectively) compared to NCI patients (70 (10) years; p<0.001) and also had fewer years of education (8 (6) and 5 (7) vs 10 (7); p<0.001). Dementia patients also differed in ethnicity (p=0.023), with a higher proportion being Malay (14% vs 3%) compared to NCI patients. They also exhibited a higher prevalence of diabetes mellitus (46% vs 16%) and hypertension (83% vs 56%) compared to NCI patients (both p<0.001). In terms of ocular characteristics, dementia patients had the thinnest RNFL (89 (18) µm) compared to CIND (90 (16) µm) or NCI patients (92 (12) µm; p=0.009).

**Table 1:** Demographics and ocular characteristics of participants with no cognitive impairment (NCI), cognitive impairment no dementia (CIND), and dementia.

Characteristics	NCI (n = 112)	CIND (n = 235)	Dementia (n = 194)	P value*
**Age (years)**	70 (10)	75 (9)	75 (10)	< 0.001
**Sex**				
**Male**	50 (45)	106 (45)	74 (38)	0.306
**Female**	62 (55)	129 (55)	120 (62)	
**Race**				
**Chinese**	103 (92)	196 (83)	151 (78)	0.023
**Indian**	5 (5)	17 (7)	12 (6)	
**Malay**	3 (3)	18 (8)	28 (14)	
**Mixed/Others**	1 (1)	4 (2)	3 (2)	
**Education (years)**	10 (7)	8 (6)	5 (7)	< 0.001
**Hyperlipidemia^1^**				
**Yes**	73 (65)	166 (71)	143 (74)	0.282
**No**	39 (35)	67 (29)	51 (26)	
**Diabetes mellitus**				
**Yes**	18 (16)	77 (33)	89 (46)	< 0.001
**No**	94 (84)	158 (67)	105 (54)	
**Hypertension^2^**				
**Yes**	63 (56)	147 (63)	160 (83)	< 0.001
**No**	49 (44)	87 (37)	33 (17)	
**Blood pressure**				
**Systolic blood pressure (mmHg)^3^**	138 (21)	142 (23)	142 (26)	0.136
**Diastolic blood pressure (mmHg)^3^**	73 (13)	75 (14)	73 (13)	0.254
**Ocular factors**				
**Spherical equivalent (diopters)^4^**	-0.8 (3.0)	-0.4 (1.9)	-0.4 (2.1)	0.051
**Signal strength of scan (0 poor to 10 good)**	8 (2)	8 (2)	8 (1)	0.294
**Average RNFL thickness (µm)**	92 (12)	90 (16)	89 (18)	0.009
**Ocular disease^5^**				
**Yes**	42 (39)	85 (38)	56 (34)	0.667
**No**	67 (61)	140 (62)	109 (66)	
**Cognitive tests**				
**MMSE total score**	28 (3)	24 (5)	16 (7)	< 0.001
**MoCA total**	26 (4)	20 (6)	12 (7)	< 0.001
**CDR global**	0 (0)	1 (1)	1 (1)	< 0.001

Data provided in median (IQR) or number (%). NCI, no cognitive impairment; CIND, cognitive impairment, no dementia; RNFL, retinal nerve fiber layer; MMSE, Mini-Mental State Examination; MoCA, Montreal Cognitive Assessment; CDR, Clinical Dementia Rating. Bold values denote statistical significance at the p < 0.05 level.

* P-values were obtained with the Kruskal-Wallis test for all continuous variables, except average RNFL thickness where mixed ANOVA was used. Chi-squared test was used for categorical variables.

1 Data from 112 NCI, 233 CIND, and 194 dementia patients.

2 Data from 112 NCI, 234 CIND, and 193 dementia patients.

3 Data from 112 NCI, 233 CIND, and 193 dementia patients.

4 Data from 105 NCI, 200 CIND, and 162 dementia patients.

5 Data from 109 NCI, 225 CIND, and 165 dementia patients.

Expectedly, dementia patients demonstrated significantly lower scores on cognitive assessments, including the MMSE and MoCA, and higher CDR global scores (p<0.001). The prevalence of ocular disease did not differ significantly across cognitive groups (p=0.667). No significant differences were observed in sex, hyperlipidemia, spherical equivalent, signal strength, systolic, or diastolic blood pressure (p≥0.051).

**Table 2:** Univariate- and multivariate-adjusted odds ratio between cognitive diagnosis and poor-quality optical coherence tomography (OCT) scans.

Characteristics	Good Quality (n = 451)	Poor Quality (n = 90)	Univariate		Multivariate^6^	
			OR (95% CI)	P value*	OR (95% CI)	P value*
**Age (years)**	74 (10)	75 (9)	1.04 (1.00 - 1.07)	0.027	1.00 (0.97 - 1.04)	0.847
**Sex**						
**Male**	204 (45)	26 (29)	*Reference*	*Reference*
**Female**	247 (55)	64 (71)	2.03 (1.24 - 3.33)	0.005	2.03 (1.15 - 3.61)	0.016
**Race**						
**Chinese**	377 (84)	73 (81)	*Reference*	*Reference*
**Indian**	30 (7)	4 (4)	0.69 (0.24 - 2.01)	0.496	0.78 (0.24 - 2.52)	0.680
**Malay**	38 (8)	11 (12)	1.50 (0.73 - 3.06)	0.271	1.16 (0.51 - 2.66)	0.726
**Mixed/Others**	6 (1)	2 (2)	1.72 (0.34 - 8.70)	0.511	2.25 (0.38 - 13.18)	0.370
**Education (years)**	8 (6)	5 (8)	0.90 (0.85 - 0.94)	< 0.001	0.95 (0.90 - 1.01)	0.126
**Hyperlipidemia^1^**						
**Yes**	317 (70)	65 (73)	1.14 (0.68 - 1.89)	0.623	-	-
**No**	133 (30)	24 (27)	*Reference*	*Reference*
**Diabetes mellitus**						
**Yes**	150 (33)	34 (38)	1.22 (0.76 - 1.95)	0.409	-	-
**No**	301 (67)	56 (62)	*Reference*	*Reference*
**Hypertension^2^**						
**Yes**	297 (66)	73 (81)	2.20 (1.25 - 3.86)	0.006	1.33 (0.70 - 2.51)	0.383
**No**	152 (34)	17 (19)	*Reference*	*Reference*
**Blood pressure**						
**Systolic blood pressure (mmHg)^3^**	141 (23)	141 (32)	1.01 (0.99 - 1.02)	0.449	-	-
**Diastolic blood pressure (mmHg)^3^**	73 (13)	72 (14)	0.99 (0.97 - 1.01)	0.482	-	-
**Ocular factors**						
**Spherical equivalent (diopters)^4^**	-0.4 (2.1)	-0.9 (2.7)	0.94 (0.85 - 1.04)	0.236	-	-
**Signal strength of scan (0 poor to 10 good)**	8 (2)	7 (2)	0.61 (0.51 - 0.72)	< 0.001	0.60 (0.50 - 0.73)	< 0.001
**Ocular disease^5^**						
**Yes**	160 (37)	23 (32)	0.78 (0.46 - 1.33)	0.369	-	-
**No**	267 (63)	49 (68)	*Reference*		-	-
**Cognitive diagnosis#**						
**NCI**	103 (23)	9 (10)	*Reference*	*Reference*
**CIND**	212 (47)	23 (26)	1.24 (0.56 - 2.78)	0.599	0.86 (0.36 - 2.05)	0.728
**Dementia**	136 (30)	58 (64)	4.89 (2.31 - 10.31)	< 0.001	2.90 (1.24 - 6.80)	0.014

Data provided in median (IQR) or number (%). NCI, no cognitive impairment; CIND, cognitive impairment, no dementia. Bold values denote statistical significance at the p < 0.05 level. Multivariate model includes cognitive diagnosis, age, sex, race, hypertension, educational years, and signal strength of scan as independent variables.

* P-values were obtained from univariate and multivariate-adjusted logistic regression respectively.

# In the multivariate model, dementia participants had a higher odds ratio (OR = 3.38; 95% CI = 1.90 - 6.02, p < 0.001) of poor scan quality compared to CIND participants. No significant difference in the likelihood of poor scan quality was observed between participants with NCI and CIND (OR = 1.17, 95% CI = 0.49 - 2.78, p = 0.728).

1 Data from 450 good-quality scans, and 89 poor-quality scans.

2 Data from 449 good-quality scans, and 90 poor-quality scans.

3 Data from 449 good-quality scans, and 89 poor-quality scans.

4 Data from 394 good-quality scans, and 73 poor-quality scans.

5 Data from 427 good-quality scans, and 72 poor-quality scans.

6 Data from 112 NCI, 234 CIND, and 193 dementia patients.

In terms of overall scan quality, 451 (83%) participants had good-quality scans, while the remaining 90 (17%) had poor-quality scans. The prevalence of good-quality scans varied significantly among the three groups: 92% of NCI participants, 90% of CIND participants, and 70% of dementia participants had good-quality scans. Table 2 shows the associations between patient demographics and overall scan quality. In the univariate regression analysis, older participants (OR = 1.04; 95% CI = 1.00 - 1.07, p=0.027), female sex (OR = 2.03; 95% CI = 1.24 - 3.33, p=0.005), individuals with hypertension (OR = 2.20; 95% CI = 1.25 - 3.86, p=0.006) and dementia (OR = 4.89; 95% CI = 2.31 - 10.31, p<0.001) were associated with increased odds of poor scan quality compared to their respective reference groups (i.e., males, individuals without hypertension, and those with NCI). Conversely, higher educational levels (OR = 0.90; 95% CI = 0.85 - 0.94, p<0.001) and greater scan signal strength (OR = 0.61; 95% CI = 0.51 - 0.72, p<0.001) were associated with lower odds of poor scan quality. No significant association was found between the presence of ocular disease and poor-quality OCT scans (p=0.369).

Multivariate analysis, adjusting for age, sex, race, hypertension, educational years, cognitive diagnosis, and signal strength of scan, revealed female sex (OR = 2.03; 95% CI = 1.15 - 3.61, p=0.016), and dementia (OR = 2.90; 95% CI = 1.24 - 6.80, p=0.014) remained associated with increased odds of poor scan quality compared to males and participants with NCI, respectively. Greater scan signal strength (OR = 0.60; 95% CI = 0.50 - 0.73, p<0.001) was associated with lower odds of poor scan quality. Of the 194 dementia patients, 42 had vascular dementia and 152 had AD. We next performed a subgroup multivariate analysis removing those with vascular dementia and found a similarly strong association between AD and having a poor-quality OCT scan (OR = 3.16; CI = 1.31 - 7.61; Supplementary Table 1).

**Table 3:** Multivariate-adjusted odds ratio between cognitive test scores and poor-quality scans. Scores from three cognitive tests were used - the Mini-Mental State Examination (MMSE), Montreal Cognitive Assessment (MoCA), and Clinical Dementia Rating (CDR).

Characteristics	Model 1#		Model 2#		Model 3#	
	OR (95% CI)	P value*	OR (95% CI)	P value*	OR (95% CI)	P value*
**Age (years)**	1.00 (0.96 - 1.04)	0.986	1.00 (0.96 - 1.04)	0.833	1.01 (0.97 - 1.04)	0.790
**Sex**						
**Male**	*Reference*	*Reference*	*Reference*
**Female**	1.81 (1.02 - 3.21)	0.041	1.73 (0.97 - 3.09)	0.062	2.01 (1.13 - 3.58)	0.017
**Race**						
**Chinese**	*Reference*	*Reference*	*Reference*
**Indian**	0.83 (0.26 - 2.63)	0.757	0.74 (0.23 - 2.38)	0.607	0.79 (0.24 - 2.56)	0.691
**Malay**	1.18 (0.53 - 2.66)	0.686	1.04 (0.46 - 2.37)	0.923	1.15 (0.50 - 2.63)	0.740
**Mixed/Others**	2.52 (0.46 - 13.79)	0.286	2.24 (0.40 - 12.65)	0.361	2.30 (0.42 - 12.68)	0.340
**Education (years)**	0.97 (0.91 - 1.03)	0.341	0.99 (0.93 - 1.06)	0.852	0.95 (0.89 - 1.01)	0.082
**Hypertension**						
**Yes**	1.52 (0.81 - 2.83)	0.194	1.45 (0.77 - 2.74)	0.252	1.40 (0.74 - 2.63)	0.297
**No**	*Reference*	*Reference*	*Reference*
**Signal strength of scan (0 poor to 10 good)**	0.62 (0.51 - 0.75)	< 0.001	0.63 (0.52 - 0.76)	< 0.001	0.62 (0.51 - 0.75)	< 0.001
**Cognitive tests**						
**MMSE total score**	0.92 (0.88 - 0.96)	< 0.001	-	-	-	-
**MoCA total**	-	-	0.90 (0.86 - 0.94)	< 0.001	-	-
**CDR global**	-	-	-	-	2.11 (1.43 - 3.10)	< 0.001

MMSE, Mini-Mental State Examination; MoCA, Montreal Cognitive Assessment; CDR, Clinical Dementia Rating. Bold values denote statistical significance at the p < 0.05 level. Multivariate model includes cognitive tests, age, sex, race, hypertension, educational years, and signal strength of scan as independent variables.

* P-values were obtained from multivariate-adjusted logistic regression.

# Data from 112 NCI, 234 CIND, and 193 dementia patients.

**Figure 2.**
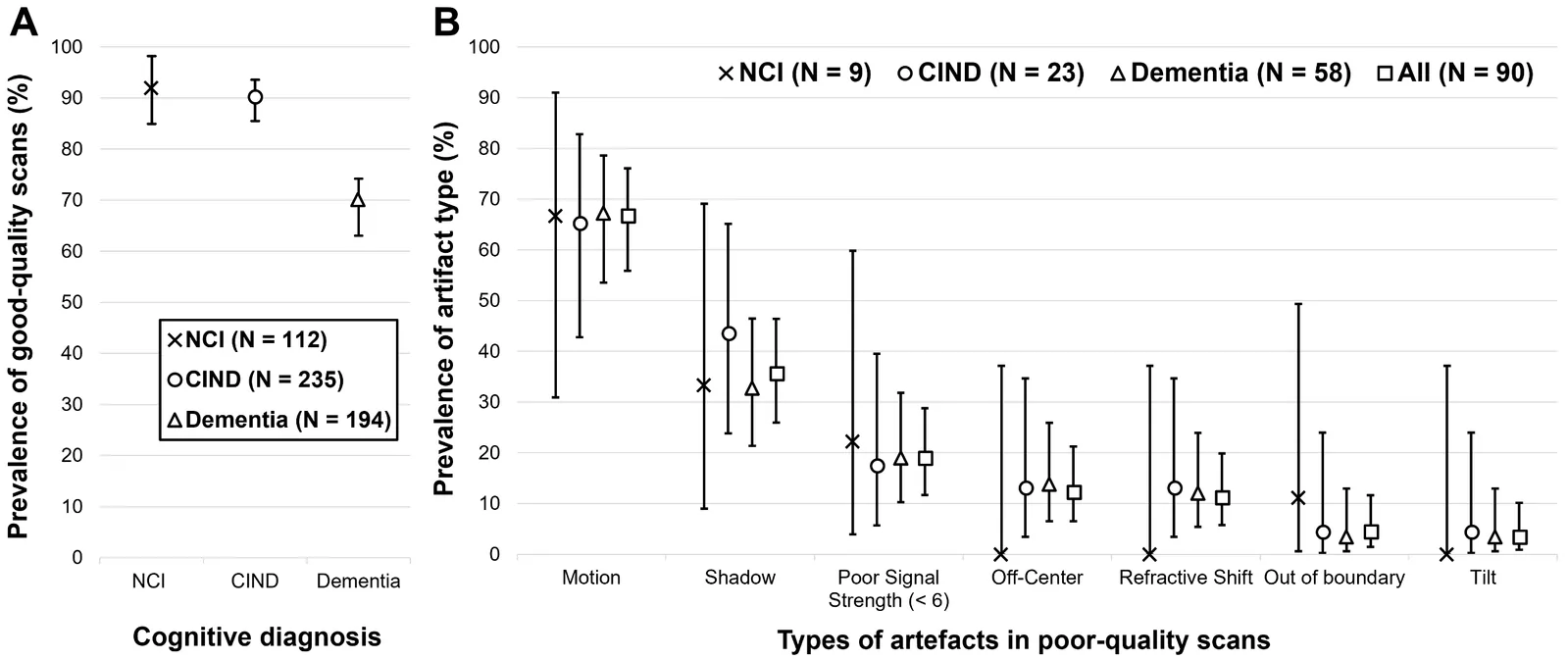
**Scan quality and artifacts in optical coherence tomography (OCT) across cognitive groups**. (**A**) Prevalence of good-quality scans varied significantly among the three cognitive groups. The dementia group (N = 194) had a significantly lower proportion of good-quality scans compared to individuals with no cognitive impairment (NCI, N = 112) and cognitive impairment no dementia (CIND, N = 235). A multivariate logistic regression analysis, adjusting for age, sex, race, hypertension, educational years, and scan signal strength, showed that patients with dementia had significantly increased odds of poor scan quality compared to those with NCI (odds ratio = 2.90; 95% CI = 1.24 - 6.80, p = 0.014). (**B**) The prevalence of artifacts in poor-quality OCT scans (N = 90) is shown, stratified by artifact type. Motion, shadows, and poor signal strength were the most commonly observed artifacts across all cognitive groups: NCI (N = 9), CIND (N = 23), and dementia (N = 58). A chi-square test was performed to compare the proportions of artifact types among cognitive groups, and no significant differences were observed: motion (p = 0.985), shadows (p = 0.655), and poor signal strength (p = 0.952). Error bars represent 95% confidence intervals.

Table 3 explores the relationship between cognitive testing scores and the likelihood of having a poor-quality scan while accounting for the influence of other factors (age, sex, race, hypertension, educational years, and signal strength of scan). Females tended to have higher odds of poor scan quality compared to males across the models, with the strongest association in Model 3 (OR 2.01; 95% CI = 1.13 - 3.58, p=0.017). Lower MMSE score (OR = 0.92; CI = 0.88 - 0.96), lower MoCA score (OR = 0.90; CI = 0.86 - 0.94), along with a higher CDR global score (OR = 2.11; CI = 1.43 - 3.10) associated with higher odds of poor scan quality (all p&<0.001). A higher signal strength was consistently associated with lower odds of poor scan quality across all models (p&<0.001). No other significant associations were observed for age, race, and hypertension. Figure 2 illustrates the prevalence of different types of artifacts found in 90 participants with poor-quality scans (n=157 individual eye scans). The most common types of artifacts observed were motion (67%), shadows (36%), and low signal strength (19%). Meanwhile, the least common types were tilt (3%), out of boundary (4%), refractive shift (11%), and off-center (12%). Generally, the proportion of motion (p = 0.985), shadows (p = 0.655), and poor signal strength (p = 0.952) were similar across the three cognitive groups (NCI, CIND, dementia).

## DISCUSSION

In this cross-sectional study, we evaluated how participants with varying degrees of cognitive impairment and performance on cognitive assessments can impact subsequent OCT scan quality and artifact prevalence. Our findings reveal a significant association between having dementia and the likelihood of producing poor-quality OCT scans. After adjusting for several confounding factors, we found that individuals with dementia, especially those with AD, were three times as likely to have a poor-quality scan compared to an NCI or CIND participant. Importantly, there was no appreciable difference in odds between the NCI and CIND cohorts regarding scan quality. To our knowledge, this study is also the first to report the strong correlation between objective cognitive assessment scores and OCT scan quality. Higher MMSE and MoCA scores were associated with better scan quality, while higher CDR scores increased the likelihood of poor scans. Taken together, our findings reveal that lower levels of cognition could have a more incremental effect on OCT scan quality, as assessed by the MMSE, MoCA and CDR.

These findings are consistent with previous research suggesting that cognitive impairment, particularly dementia, can significantly affect the quality of OCT scans. Our findings align with previous research demonstrating that difficulties in maintaining fixation, impaired ocular motility, and decreased visual attention in dementia patients contribute to poor-quality OCT images [22]. Studies have shown higher rates of ungradable OCT angiographic images and poorer quality in dementia patients, with lower cognitive scores directly linked to scan quality [6]. However, the relatively small proportion of dementia patients (n=23) in their study may have limited statistical power, as cognitive scores became nonsignificant in the multivariate analysis. Marquié et al. also observed a similar pattern in a biracial cohort, where dementia patients were more likely to have poorer image quality than those with subjective cognitive decline (SCD) and MCI [23]. Our study extends these findings by adjusting for a wider range of confounding factors, such as age, sex, race, hypertension, educational years, and OCT signal strength. This strengthens the association between cognition and OCT scan quality, highlighting the importance of considering cognitive factors when interpreting OCT results in patients with neuro-degenerative diseases.

Our findings indicate that women were more susceptible to poorer OCT scan quality compared to men. While the exact cause is yet unknown, biological factors or cultural norms associated with sex could play a role in this outcome. Biological differences, such as cultural biases in healthcare and clinical decision-making [24-26] could influence how women are treated and assessed during OCT examinations. Further research is needed to elucidate the specific factors and pathways involved in this sex-related effect.

As expected, lower signal strength was significantly associated with higher odds of poor scan quality. Our findings are consistent with previous studies that demonstrated decreased signal strength affects the reliability of various OCT measurements [27-30], including vessel density for OCTA systems [31], RNFL thickness using Stratus OCT devices [32], and artifact frequency using Triton OCTA devices [33]. However, our study extends these findings by validating the association in a different OCT device and a larger cohort with diverse ocular pathologies and cognitive diagnoses.

Our analysis of artifact types in OCT scans identified motion as the most prevalent artifact (68%), followed by shadows (34%), and low signal strength (19%). While no prior studies have specifically examined artifact types in OCT scans, our findings align with existing literature on OCTA scan artifacts. Abraham et al. reported low signal strength (66%) and motion (22%), as the primary causes of poor OCTA scan quality [6]. Similarly, Holmen et al. identified artifacts in 97% of OCTA images, with the highest individual prevalences being motion (93.1%), defocus (74.9%), followed by shadow (62.3%), and tilt (50.5%) [17]. The higher prevalences observed by Holmen et al. may be attributed to the more stringent requirements of OCTA scanning, which is more sensitive to movement and requires greater patient cooperation due to its angiographic nature. Notably, the proportion of artifact types was similar among the NCI, CIND, and dementia groups, suggesting that while cognitive impairment can increase the likelihood of poor-quality scans, the specific types of artifacts observed are not significantly different across these groups.

### Clinical implications

Patients with AD are at increased risk for ocular pathologies like moderate diabetic retinopathy [5], and open-angle glaucoma [34]. The higher prevalence of artifacts in OCT scans from AD patients can hinder the acquisition of good-quality scans, potentially delaying or affecting the accuracy of diagnosis and monitoring of eye diseases in this population. To improve the reliability of OCT scans in patients with neurodegenerative diseases, strategies such as providing clear instructions during the image acquisition process should be implemented [35]. Additionally, our findings suggest that difficulty in cooperating with imaging procedures, such as maintaining fixation and following instructions, subsequently leading to poor scans, may inadvertently serve as an early indicator of cognitive impairment.

Performing reliable OCT scans on dementia patients requires tailored strategies to address the unique challenges posed by cognitive decline. A combination of simplified instructions, proper staff training, and active involvement from caregivers can facilitate communication and improve patient cooperation. Reducing distractions, such as patching the fellow eye and minimizing sensory disturbances (e.g., loud noises, bright lights), helps to alleviate anxiety and agitation. Shorter scaning protocols with frequent breaks are beneficial for maintaining the patient’s attention span, while scheduling scans early in the morning may mitigate challenges associated with sundowning [36-38]. Advanced OCT technologies, such as automated eye-tracking and motion artifact correction, can help to compensate for unstable fixation or minor head movements. Proper staff training in communication techniques and dementia care further enhances scan reliability. These strategies ensure higher scan quality while prioritizing patient comfort and tailoring care to each individual’s needs.

Leveraging advancements in OCT technology also mitigates the impact of dementia on OCT scan quality and subsequent diagnoses. For instance, deep learning (DL) algorithms are being actively developed to improve diagnostic performance in OCT images, achieving increasingly high sensitivities, specificities, and areas under the curve (AUC) [39-41]. However, many of these algorithms are primarily trained on high-quality scans [42, 43], limiting their performance on lower-quality scans [44-46]. To address this, a significant focus should be placed on improving image quality through hardware and software advancements. Improvements in OCT technologies, such as those proposed by Tan et al., who combined megahertz swept source laser with improved post-processing to reduce scan acquisition time and motion correction [47, 48], can help mitigate issues related to scan quality, particularly motion artifacts, which were the most common problem observed in this study.

### Strengths and limitations

Key strengths include a standardized and well-validated methodology, including rigorous technician training, objective quality control, neurocognitive testing, and clinical assessments. Our study benefited from a large multiethnic Asian cohort with near-equal representation from the three groups (NCI, CIND, dementia), which bolsters the statistical confidence of our results. This allowed for accurate adjustment of potential confounders such as age, sex, race, hypertension, educational level, and signal strength. Another strength is the relatively large sample size with fair representation from the three cohorts involved. However, the study may not be fully generalizable to all Asian populations due to the potential cultural differences that could influence communication styles and attitudes towards medical procedures. Additionally, the recruitment process may have introduced biases, as participants were recruited from memory clinics rather than randomly sampled from the general population.

## Conclusions

This study on a multiethnic Asian population demonstrated that dementia patients, especially those with AD, were significantly more likely to have poor-quality OCT scans. Patients with poorer cognition as assessed by MMSE, MoCA, and CDR, along with female patients, were also at higher risk. Physicians should pay particular attention to these patients who may face challenges during the scanning procedure and intervene appropriately. Addressing these barriers to care can improve vision outcomes for vulnerable individuals.

## Supplementary Materials

The Supplementary data can be found online at: www.aginganddisease.org/EN/10.14336/AD.2024.1744.
