# Supplementary material for: Association Between Dementia and Optical Coherence Tomography Scan Quality
Source: Aging Dis. 2025 Feb 13;17(1):566–77. doi: 10.14336/AD.2024.1744 (PMC12727133; doi:10.14336/AD.2024.1744)
Supplement: Supplementary file 1 — The Supplementary data can be found online at: www.aginganddisease.org/EN/10.14336/AD.2024.1744. [file AD-17-1-566-s.pdf]

SUPPLEMENTARY DATA

Association Between Dementia and Optical Coherence Tomography Scan Quality

Reuben Jyong Kiat Foo, Damon Wong, Nur Fidyana Binte Abdul Gani, Bingyao Tan, Munirah Binte Ismail, Gerhard Garhöfer, Laetitia Hinterhuber, Narayanaswamy Venketasubramanian, Christopher Li-Hsian Chen, Leopold Schmetterer, Jacqueline Chua

© 2025. Foo RJK et al. Published online at http://www.aginganddisease.org/EN/10.14336/AD.2024.1744

SUPPLEMENTARY DATA

Supplementary Table 1. Multivariate-adjusted odds ratio for poor-quality scans with cognitive diagnosis, amongst NCI, CIND, and AD subjects

Characteristics	Good Quality (n = 418)	Poor Quality (n = 81)	Univariate		Multivariate[{]	
			OR (95% CI)	P value	OR (95% CI)	P value
Age (years)	74 (10)	75 (10)	**1.04 (1.01 – 1.08)**	**0.013**	1.01 (0.97 – 1.05)	0.721
**Sex**						
Male	181 (43)	22 (27)	*Reference*		*Reference*	
Female	237 (57)	59 (73)	**2.05 (1.21 – 3.47)**	**0.007**	1.81 (0.97 – 3.39)	0.065
**Race**						
Chinese	351 (84)	67 (83)	*Reference*		*Reference*	
Indian	29 (7)	2 (3)	0.36 (0.08 – 1.55)	0.171	0.45 (0.10 – 2.09)	0.310
Malay	32 (8)	10 (12)	1.64 (0.77 – 3.49)	0.202	1.32 (0.54 – 3.22)	0.538
Mixed/Others	6 (1)	2 (3)	1.75 (0.35 – 8.84)	0.500	2.18 (0.36 – 13.05)	0.394
Education (years)	8 (7)	5 (8)	**0.90 (0.85 – 0.95)**	**< 0.001**	0.95 (0.90 – 1.02)	0.144
**Hyperlipidemia**[†]						
Yes	287 (69)	58 (73)	1.19 (0.70 – 2.03)	0.514	-	-
No	130 (31)	22 (28)	*Reference*		*Reference*	
**Diabetes mellitus**						
Yes	130 (31)	30 (37)	1.30 (0.79 – 2.14)	0.296	-	-
No	288 (69)	51 (63)	*Reference*		*Reference*	
**Hypertension**[‡]						
Yes	264 (64)	64 (79)	**2.17 (1.23 – 3.84)**	**0.008**	1.38 (0.72 – 2.63)	0.331
No	152 (37)	17 (21)	*Reference*		*Reference*	
**Blood pressure**						
Systolic blood pressure (mmHg)[§]	141 (23)	142 (31)	1.01 (0.99 – 1.02)	0.478	-	-
Diastolic blood pressure (mmHg)[§]	73 (13)	72 (16)	0.99 (0.97 – 1.01)	0.455	-	-
**Ocular factors**						
Spherical equivalent (diopters)[‖]	-0.5 (1.9)	-0.9 (2.8)	0.94 (0.85 – 1.04)	0.210	-	-
Signal strength of scan (0 poor to 10 good)	8 (2)	7 (2)	**0.60 (0.50 – 0.72)**	**< 0.001**	**0.59 (0.48 – 0.73)**	**< 0.001**
**Cognitive diagnosis***						
NCI	103 (25)	9 (11)	*Reference*		*Reference*	
CIND	212 (51)	23 (28)	1.24 (0.56 – 2.78)	0.599	0.83 (0.35 – 2.01)	0.685
AD	103 (25)	49 (61)	**5.44 (2.54 – 11.66)**	**< 0.001**	**3.16 (1.31 – 7.61)**	**0.010**

Data provided in median (IQR) or number (%).

NCI, no cognitive impairment; **CIND**, cognitive impairment, no dementia; **AD**, Alzheimer's disease.

Bold values denote statistical significance at the $p < 0.05$ level.

Multivariate model includes cognitive diagnosis, age, gender, race, hypertension, educational years, and signal strength of scan as independent variables.

© 2025. Foo RJK et al. Published online at http://www.aginganddisease.org/EN/10.14336/AD.2024.1744

SUPPLEMENTARY DATA

*In the multivariate model, dementia participants had a higher odds ratio (OR = 3.79; 95% CI = 2.08 – 6.90, $p < 0.001$) of poor scan quality compared to CIND participants. No significant difference in the likelihood of poor scan quality was observed between participants with NCI and CIND (OR = 1.20, 95% CI = 0.50 – 2.88, p = 0.685).

[†]Data from 417 good-quality scans, and 80 poor-quality scans.

[‡]Data from 416 good-quality scans, and 81 poor-quality scans.

[§]Data from 416 good-quality scans, and 80 poor-quality scans.

[||]Data from 370 good-quality scans, and 66 poor-quality scans.

[{]Data from 112 NCI, 234 CIND, and 151 AD patients.

© 2025. Foo RJK et al. Published online at http://www.aginganddisease.org/EN/10.14336/AD.2024.1744
